# Fundamental Clock of Biological Aging: Convergence of Molecular, Neurodegenerative, Cognitive and Psychiatric Pathways: Non-Equilibrium Thermodynamics Meet Psychology

**DOI:** 10.3390/ijms23010285

**Published:** 2021-12-28

**Authors:** Victor V. Dyakin, Nuka V. Dyakina-Fagnano, Laura B. Mcintire, Vladimir N. Uversky

**Affiliations:** 1 The Nathan S. Kline Institute for Psychiatric Research (NKI), 140 Old Orangeburg Road, Bldg, 35, Bld. 35. Rom 201-C, Orangeburg, NY 10962, USA; 2 Child, Adolescent and Young Adult Psychiatry, 36 Franklin Turnpike, Waldwick, NJ 07463, USA; doctor@nikadyakina.com; 3 Department of Pathology and Cell Biology, Institute for Research on Alzheimer’s Disease and the Aging Brain, Columbia University Medical Center, New York, NY 10032, USA; lbm2110@cumc.columbia.edu; 4 Department of Molecular Medicine and Byrd Alzheimer’s Research Institute, Morsani College of Medicine, University of South Florida, 12901 Bruce B. Downs Blvd., MDC07, Tampa, FL 33612, USA; vuversky@usf.edu

**Keywords:** spontaneous, non-enzymatic, post translational modifications, racemization, biological clock, natural selection, allostatic load, psychological aging, psychological stress, stress response system, phase transitions, racemization theory of aging

## Abstract

In humans, age-associated degrading changes, widely observed in molecular and cellular processes underly the time-dependent decline in spatial navigation, time perception, cognitive and psychological abilities, and memory. Cross-talk of biological, cognitive, and psychological clocks provides an integrative contribution to healthy and advanced aging. At the molecular level, genome, proteome, and lipidome instability are widely recognized as the primary causal factors in aging. We narrow attention to the roles of protein aging linked to prevalent amino acids chirality, enzymatic and spontaneous (non-enzymatic) post-translational modifications (PTMs ^SP^), and non-equilibrium phase transitions. The homochirality of protein synthesis, resulting in the steady-state non-equilibrium condition of protein structure, makes them prone to multiple types of enzymatic and spontaneous PTMs, including racemization and isomerization. Spontaneous racemization leads to the loss of the balanced prevalent chirality. Advanced biological aging related to irreversible PTMs ^SP^ has been associated with the nontrivial interplay between somatic (molecular aging) and mental (psychological aging) health conditions. Through stress response systems (SRS), the environmental and psychological stressors contribute to the age-associated “collapse” of protein homochirality. The role of prevalent protein chirality and entropy of protein folding in biological aging is mainly overlooked. In a more generalized context, the time-dependent shift from enzymatic to the non-enzymatic transformation of biochirality might represent an important and yet underappreciated hallmark of aging. We provide the experimental arguments in support of the racemization theory of aging.

## 1. Introduction

“*The roots of stress research lie in the belief that stress can accelerate biological aging*”

[1]

Molecular psychology and psychiatry represent an emerging modern research trend. However, the role of prevalent bio-molecular chirality and molecular aging in the function of the stress response system (SRS) is practically neglected. Indeed, the consequences of psychological stresses linked to biological aging are evident at the organism, system, cellular, and molecular levels [2,3]. Under stress conditions, self-perception is contributed to by the sense of embodiment [4] and by conscious control of your own thoughts [5].

No doubt, the age-dependent biological processes are influenced by the subject’s state of mind and psychological state [6,7]. The functioning of an organism at the molecular and cellular levels underlies humans’ perception of environmental challenges, self-perception, and age-associated modification of physiological and cognitive functions. No doubt that all hierarchical domains of biological events—including molecular–molecular, molecular–cellular, cellular–organismic, organismic–cognitive/behavioral—exhibit bi-directional impact [6,8]. The understanding of such bidirectionality constitutes the solid ground for pharmacological and psychological treatment. From the thermodynamic perspective, the organism, as a whole, as well as its major constituents (biomacromolecules, cellular organelles, cells, tissues, and organs) maintains themselves in the entropy-driven non-equilibrium state [9,10,11].

We will focus on the thermodynamics of protein folding in the age-dependent association with the function of the stress response system (SRS), and provide the experimental arguments in support of the racemization theory of aging.

### 1.1. Aging, Entropy, and Aging Defense System

“*It is by avoiding the rapid decay into the inert state of ‘equilibrium’ that an organism appears so enigmatic*”

[9]

Conceptualization of the underlying mechanisms of aging is in high demand due to many age-related diseases. Significant progress is made from the view that the variable transient fluctuating entropy (from the Greek entropia = transformation) in the non-equilibrium (NE) state of a biomolecular ensemble is under permanent impact by the thermodynamic tendency toward the high-entropy state of equilibrium. NE thermodynamic of living systems is closely associated with the concepts of biochirality, entropy, biological information processing, and aging [12]. NE thermodynamic theories, in particular, Classical Irreversible Thermodynamics (CIT) [13], complemented by the concept of fluctuating entropy [14,15], provide a valuable formalism for understanding the dynamics of living systems, including the origin of life, cell differentiation, as well as the synthesis of the homochiral population of proteins and the spontaneous loss of conformational entropy during folding [16,17,18].

Energy-consuming ribosomal protein synthesis (the human body requires production of 1021 ATP molecules per second) continuously creates the pool of homochiral; high entropy unfolded biomolecules in the thermodynamically NE state. Prevalent chirality of nascent proteins is transferred to all levels of their structural hierarchy during spontaneous condensation (folding) to native (i.e., functional) state (NS) [19,20]. The relaxation, conformation, and formation of protein assemblies constitute large many-body systems that operate in a fluctuating, out-of-equilibrium environment.

Changes in the residual conformational entropy at the protein level are significant components of the thermodynamics of folding, binding, enzyme-substrate recognition, and time-dependent protein dysfunctions [21,22]. Spontaneous protein folding (3-D transformation) to NS is accompanied by free energy loss and reduction of configurational entropy.

**Figure 1:**
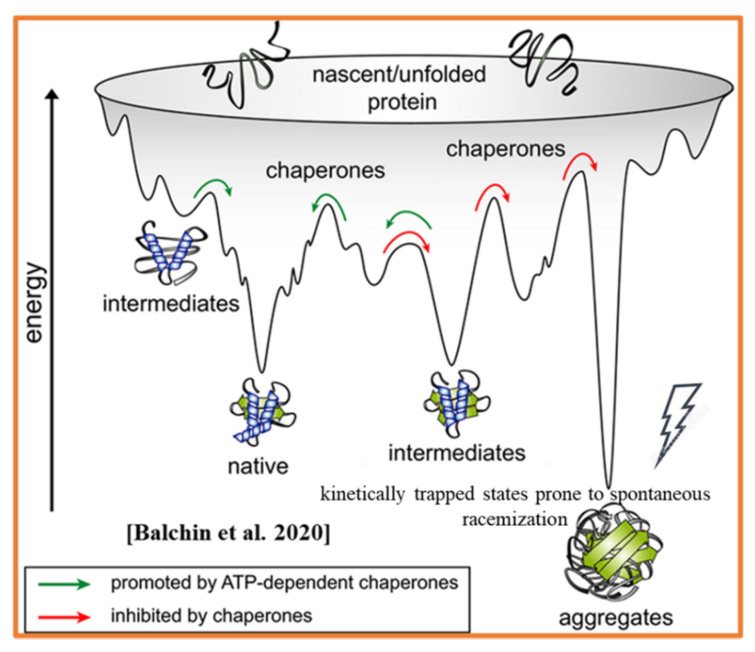
Funnel-shaped potential free-energy landscape. Impact of spontaneous racemization. Adopted with alterations from [25].

NS of homochiral proteins (trapped in a local energy minimum) is thermodynamically only relatively stable and, therefore, is prone to further spontaneous relaxation to aberrant conformation and aggregative states. Such spontaneous processes can be triggered by amino acid (AAs) racemization [23,24]. Spontaneous racemization is known as one of the unavoidable degrading forces of the functional state of proteins. More (than native) thermodynamically stable states of proteins such as amyloids, fibrils, and aggregates are associated with protein aging translated to the cellular and organism levels. However, even recent advances in the understanding of protein folding and miss-folding ignore the homochirality of the NS and, correspondently, overlook the contribution of spontaneous racemization to protein aging [25] (Figure 1). Furthermore, protein aggregation is known to slow down the rates of structural conversions (leading to kinetic trapping), extend the cellular lifetime of trapped protein, and make them prone to the impact of spontaneous (molecular environment-specific) racemization.

In terms of quantitative thermodynamic variables, spontaneous protein fold from the initial chain of AAs to the NS is accompanied by the loss of Gibbs energy (ΔG) and minimizing the entropy (ΔS ^Fold^). From the thermodynamic perspective (1), some other processes should compensate for the loss of Δ S ^Fold^.

ΔG = ΔH − T × ΔS(1)

and

S = k × ln(W)(2)

where k is Boltzmann’s constant and W is the number of microstates that give rise to the macro-state of interest.

One of them is the gain of enthalpy under forces of the binding (condensation). Two others are the increase in the entropy of solvent and increment in the entropy of protein due to loss of protein homochirality (racemization) [9]. Therefore, aging can be considered as an exchange between the accumulation of damage and compensatory mechanisms. The complexity of compensatory events comprises the aging defense mechanism, which, unfortunately, also is vulnerable to the impact of spontaneous biological reactions. Age-associated degrading changes are observed in psychological and neurobiological processes underlying the decline in basic functions of the organism, including spatial navigation [26,27] and time processing [28], contributing to the perception of the virtual reality environment [29]. Evolutionary biologists conclude that the force of selection declines as a function of age due to the decreased likelihood of reproduction. The fact that the force of selection declines as a function of age promotes the appearance of two main hypotheses formulated to explain why organisms age: the mutation accumulation (MA) and the antagonistic pleiotropy (AP) hypotheses [30,31,32,33]. MA theory proposed by Peter Medawar in 1952 [34] suggests that mutations with deleterious late-life effects can accumulate if such products are confined to late life, when selection against them is weak. Williams AP theory proposed by George C. Williams in 1957 [35] is based on the fitness trade-offs and assumes the ability of a gene to control more than one trait, with part of these traits being beneficial to the fitness of the organism early on in life, whereas other traits are detrimental to the fitness later on.

The racemization hypothesis of aging (RHA) helps integrate the evolutionary and molecular genetics of aging. Experimental evidence suggests that both aging and psychological stress affect the immune, hormonal, and neuro-transmitting systems [36,37,38,39]. Stress is recognized as a fundamental physiological phenomenon essential to survival and related to several psychiatric [35] and neurodegenerative [40] disorders. A common view that the response to stressful stimuli is triggered and exacerbated by the SRS that integrates a wide diversity of brain structures, neuronal circuits, as well as perceptual and cognitive functions. In other words, the mechanisms of stress response integrate brain and body activity at the molecular, cellular, and neuronal network levels. At the molecular level, the functions and age-related decline of the STS are linked to the maintenance or deterioration of the prevalent chirality of proteins. Protein aging is a trade-off between the maintenance of prevalent chirality and spontaneous racemization. From this integrative view, it is evident that the STS mediates the physiological and psychological outcomes of aging individuals.

### 1.2. Psychological and Physical Stressor

**Figure 2:**
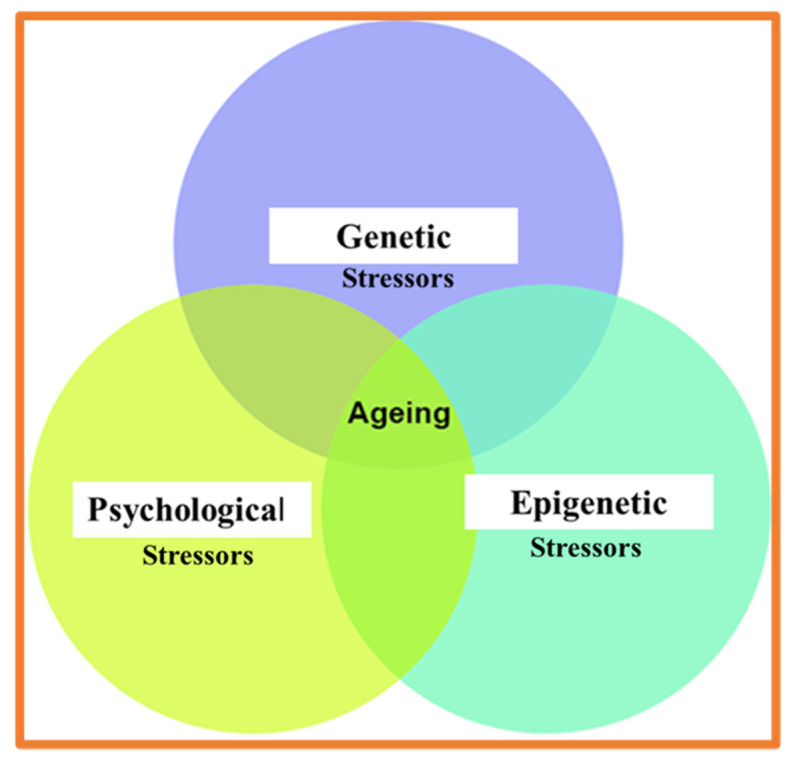
Major determinant of biological aging.

The discrimination of psychological and physical stressors is essential for understanding the complex mechanism of the human SRS (Figure 2) [41,42,43]. Psychological stressors are defined as stimuli that threaten the current state and are perceived in an anticipatory condition, e.g., aversive environmental stimuli, predator-related cues, and failure to satisfy internal drives. Adult diseases [44] and accelerated aging [45] are often associated with acute stressors and the developmental, biological, and psychological abnormalities occurring in childhood. Epigenetic mechanisms of physical and psychological stressors make them become biologically embedded into physiology (at molecular, cellular, systemic, and organism levels) [46,47,48]. The epigenetic mechanisms of physical and psychological stressors relate how environmental factors are translated to physiological events through sensory perception, cognitive, and emotional abilities. At the protein level, the stress response occurs through the system of enzymatic and non-enzymatic (spontaneous) PTMs (PTMs ^SP^). Spontaneous and stress-induced aging of proteins is a complex process of nonenzymatic chemical reactions which contribute to the range of metabolic diseases [49,50] Most studied age-associated non-enzymatic PTMs contributing to protein aging include oxidation, nitration, glycation, and racemization. We will be primarily focused on irreversible spontaneous racemization [51,52].

### 1.3. Biochirality, Spontaneous Reactions and Aging

“*Chirality is a fundamental, persistent, but often overlooked feature of all living organisms on the molecular level as well as on the macroscopic scale*.”

[53] (See also [54,55])

At the molecular level, aging is associated with three primary classes of bio-molecules prone to spontaneous modifications: (1) nucleotides (NTs) within DNA sequence, (2) amino acids (AAs) within peptides and proteins and (3) lipid content of the membrane bilayers. In these cases, the irreversibility of reactions results in the time-dependent accumulation of abnormal molecular complexes. Both NT and AA molecular pathways of aging are closely interconnected [56] and obey the laws of non-equilibrium thermodynamics. The influx of energy maintains the system of molecular objects in an asymmetric non-equilibrium dynamic steady state, which can retain their asymmetry (homochirality) for a time longer than the time of spontaneous racemization [57]. Meaning that any compromise of energy supply will open a window to spontaneous racemization. Spontaneous DNA mutations arise from a variety of sources, including irreversible changes of NTs sequence, leading to the errors in DNA replication.

Spontaneous alterations in DNA structure result in chaining the functions implicated in protein synthesis, cell proliferation, cell polarity and organism morphology (including bilaterality). PTMs ^Sp^ of proteins, the second causal agent of organism aging, will be the focus of our attention here. Spontaneous reactions such as isomerization, epimerization, and racemization taking place in long-lived protein (LLPs), have two physiologically essential pathogenic consequences: prevention of proteolytic degradation [58] and non-enzymatic cleavage [59]. The evolution of living organisms has established mechanisms to exclude D-amino acids (D-AAs) in their protein synthesis machinery to maintain the prevalent molecular chirality and stereo-specific catalysis (three major sources of D-AAs in mammals are intrinsic racemization (enzymatic and spontaneous), diet, and gut microbiota). However, recent development [60,61,62] shows that this mechanism has not absolute prevalence. For example, a small probability exists for ribosomal incorporation of D-AAs into AAs sequence of peptides and protein remains. In addition to the ribosomal source, D-AAs are created by the mechanism of enzymatic and spontaneous post-translational modifications (PTMs). Small amount D-AAs in an organism signify the evolutionary selected delicate balance between two isomers, suggesting that any disturbance in this balance can be harmful. It is notable that D-AA-containing peptides and proteins demonstrate two distinct features: (1) resistance toward proteases and association with prolonged half-lives (2) [60,62]. Biochirality, evident in the pathways of enzymatic and spontaneous racemization of AAs and PTMs of proteins, is the emerging as a critical and yet experimentally and theoretically challenging topic. The attention to spontaneous modifications of free and peptide/protein-incorporated AAs becomes essential due to their role.

In protein aging, cell physiology, and neuropathology. Spontaneous chemical reactions, such as racemization, occur by various pathways, including oxidation, cyclization, and elimination reactions. Spontaneous racemization is a critical determinant of thermodynamically irrevocable (i.e., aberrant) protein folding, responsible for the side products of biochemical reactions. Many studies are devoted to the consideration of transitions from tetrahedral to planar electron configurations. Planar intermediates are critical determinants of the racemization barrier and rate of racemization [63]. Free AAs and peptides/protein-incorporated AAs are characterized by significantly different rates of spontaneous reactions. Due to the complexity of three-dimensional structure of globular proteins and the presence of many functional groups, susceptibility to spontaneous or enzyme-dependent stereo-chemical modifications is much higher for peptides and proteins than for free AAs [63]. Therefore, future research in this field will enormously contribute to understanding the molecular mechanism of protein misfolding, aggregation, disfunction, degradation, and aging.

The chain of chirality transmissions across the length scales and level of organization is recognizes as a fundamental feature of all living creatures. The prevalent molecular handedness guides the chirality of the cells, while cell chirality drives the left/right asymmetric development of individual organs and organisms [49,51,55]. Contemporary concepts of prevalent biochirality and virtual reality brings the new dimensions to the exploring mutual influence of biological and cognitive domains of self [4,5,64,65,66,67] and new meaning to Schopenhauer’s view on the world. As the manifestation of the “Will and Representations”. Our review aims to attract attention toward the multidisciplinary field of biological chirality (or biochirality) and its broad-reaching implications at both the molecular and organism levels. As a branch of natural science, the concept of biochirality has broad implications in multiple fields ranging from DNA function to protein synthesis, neurotransmission, and bilaterality of cognitive functions. The rationale of such an attempt is that integration and synthesis of diverse, dispersed, and unsystematic experimental facts are required for better understanding of the fundamental nature of complex phenomena. Congruent advances in biochemical and biophysical studies provide a common framework for establishing a new level of academic research, medical treatments, and drug design.

To advance the field, it is necessary to consider the chronology of the ideas reflecting the progress in understanding the biochirality-related causality of aging phenomena. It is clear, that there are endogenous and exogenous stressors (agents) continuously challenging the integrity of DNA, transcriptome, and proteome, resulting in progressive aging of the organism, but the main causal triggers, at the molecular level, remains unknown. To better understand the impact of biochirality on the field of aging research, we will briefly review here the chronology of major developments regarding the biochirality-related causality of aging. 

In 1978, Poplin and DeLong proposed the hypothesis that aging can be accelerated due to the enzymatic racemization [68]. In 1994, Fujii and colleagues concluded that “racemization, isomerization, and oxidation of αB-crystallin occur spontaneously in the aging process” [69], and Mori pronounced the biological significance of racemization in the neuropathogenesis of Alzheimer’s disease (AD) [70]. In 1995, John discussed the effect of aging on the turnover of muscle proteins [71]. The crucial role of non-enzymatic PTMs of proteins in aging was recognized in the previous century [72]. Later Maddox, in his “The Encyclopedia of Aging”, noticed that the “extend to which racemization contribute to the harmful consequence of aging remains uncertain” [73]. This conclusion indicates that the impact of racemization as a causal in aging was at that time, not yet clearly understood.

In 2002, Ritz-Timme & Collins linked the “natural” aging of proteins with the autonomic racemization of long-lived proteins (LLPs) [74]. In 2014, Inoue concluded that the most convenient biomarkers of protein aging were the spontaneous racemization of AAs [75]. Currently, many studies emphasize the existence of the abnormal age-related translational and post-translational protein homeostasis associated with spontaneous forms of PTMs (such as oxidation and oxidative phosphorylation) [75]. It is also recognized that progressive deterioration in the ability of the cells to preserve the stability of their proteome occurs with age, even in the absence of disease, and it likely contributes to different aspects of “natural” aging. However, the principal role of spontaneous racemization in geriatric science remains overlooked. Based on the above evidence, it is reasonable to conclude that spontaneous racemization (as a specific form of PTMs) may be a valuable molecular biomarker of age-associated neurodegenerative and psychiatric disorders.

## 2. Crosstalk of Physiological and Psychological States

### 2.1. Molecular Level

Many studies emphasize the bidirectional relationships between the psychological and physiological states of the individual. Molecular and cellular consequences of psychological stress in human aging are widely documented [2] but poorly understood. The prevalence of specific stereoisomers is the distinct and crucial feature of the molecular determinants underlying the crosstalk between the physiological and psychological pathways. However, the specificity of psychological states and the role of molecular chirality in above indicated bidirectional link is not widely discussed and should be reconsidered in view of new results. The inference to the crucial link between physiological and psychological status of the individual requires consideration of the principals of biochirality. The human’s mental state’s development, integrity, and decline are mediated by the multifactorial interplay of genetic and environmental factors at their convergence with the molecular domains (Figure 1 and Figure 2). Several types of biologic macromolecules, including and lipids, exhibit two interactive (co-existing) characteristics. First–prevalent chirality [68] and, second, vulnerability to multiple types of aberrant modifications [56,76]. Prevalent molecular chirality DNA, RNAs, proteins, results from natural selection (NS), providing the functionality of DNA, RNAs, proteins, and lipids along with perceptual and cognitive abilities to live organisms. Biomolecular assembly under condition of prevalent chirality is a key driving force in all life processes [77]. However, several biomolecular structures, including amyloids pathological amyloids and amyloid-like aggregates, are known as the by-products of the main evolutionary pathway. Aberrant malfunctional structures containing D-AAs are the main contributors to the dysregulation of neuronal signaling, biological aging, and development of various pathological conditions [78]. Aging, as manifested in the neurodegenerative and psychiatric disorders, is associated with the progressive decline of functions at the molecular, cellular, organ, and organism levels [75,76,77,78,79]. At the molecular level, aging is associated with the impact of physical, environmental, and psychological stressors [41,43,45,64,65,66,67,68,69,71,72,77,78,79,80,81,82,83,84,85,86,87,88,89]. Acute and chronic psychological stressors are characterized as complicated constructs capable of inducing the modifications at DNA and protein levels (epigenetically/environmentally induced genes are silenced or activated via hypermethylation, which causes transcriptional and epigenomic changes in response to stress) [89,90,91,92,93,94,95]. In particular, the neuropathology of schizophrenia is characterized by the convergence of morphological, cellular, and molecular correlates [91,92,93,94,95]. The most common pathways of schizophrenia and neurodegeneration include abnormalities in glutamatergic and dopaminergic neurotransmission, altered synaptic plasticity, and elevated brain level of amyloid-β (A-β) [96,97]. 

During the last decade, the reciprocal influences of biology, psychology, behavioral, and social factors on health and illness gain significant attention. But understanding of the effect of psychological stressors on spontaneous biological events is currently in the embryonic state [98,99]. It is known that people exposed to major psychological stressors in early life, exhibit elevated rates of mortality from chronic diseases of aging [100]. The impact of psychological stressors (PSs) on spontaneous, age associated PTMs of protein remains unexplored and unexplained. In our view, the critical mechanism underlying the link between PSs and lifespan is spontaneous PTMs including racemization.

Genetic, epigenetic, and psychological determinants (pathways) of biological aging. The most common contributing factor, at the molecular level, is protein aging, i.e., spontaneous racemization (see Figure 1).

### 2.2. System Level

In higher animals, including humans, the biological adaptation to stressful stimulus involves co-activation of the neural, endocrine, and immune systems [100,101,102,103]. All three are highly integrated components of the SRS and naturally share many fundamental aspects of neurobiology associated with the chirality transfer from the molecular to the system level. The endocrine and immune systems exhibit age-related alterations [3,104] and the asymmetry in the activity of corresponding brain regions have a long history of experimental observation [105]. Still, their relation to protein homochirality, spontaneous PTMs, and left/right brain asymmetry is not sufficiently understood even though the link between two poles of this phenomenon–morphological bilaterality/handedness [106,107,108] and prevalent molecular chirality [82,109,110,111,112] gain significant advances in recent decades. The association of the SRS with the physiological and pathological molecular chirality is the subject of our specific attention. The molecular and cellular consequences of psychological stress in humans are considered essential determinants of brain functional laterality [83,107] and aging [52,112,113]. However, the fundamental causations of molecular aging following (associated with) psychological stress are poorly understood. Therefore, translating psychological stress into the consequence of bio-molecular events remains challenging task.

### 2.3. Organism Level

At the organism level, the age-related decline in cognitive function includes compromised memory, language, critical thinking, and decision-making. Impaired cognition affects the psychological-behavioral functions, including social communicants and self-perception, leading to age associated psychological stress linked with a range of psychopathologies (including compromised memory and allostatic load). Psychological stress, in turn, is a significant determinant of cognitive decline. Allostery is a fundamental mechanism by which proteins respond to environmental cues.

For example, hormonal activation of enzyme activity is highly stereo-specific reactions (allosteric modulation) that link stereochemistry of protein synthesis, function, and degradation with the stress-induced activation of spontaneous PTMs [114]. Stress-induced, age-associated deterioration of cognition and psychological functions correlates with neuronal degeneration, misfolding, disfunction, and aggregation of synapse-associated proteins and peptides, including NMDA receptors, A-β, TAU, neuroligins, neurexins, and many others [97]. An emerging concept proposes that molecular and sub-cellular structures “sense, integrate, and transduce psychosocial and behavioral factors”, suggesting the reciprocal causation between age-associated molecular, cognitive and psychological factors [55,78,99,100,107,108,109,110,111,112,113,114,115,116,117,118,119,120,121]. The psychological perspective of aging, seeing through molecular and cell biology prism, helps better understand the complex interrelationships among protein folding, cell signaling, brain structure, cognition, and behavior for both healthy and pathological trajectories [122,123].

### 2.4. Chiral Psychotherapy

L-carnitine (β-hydroxy-γ-N-trimethylaminobutyric acid) is necessary for energy production and fatty acid metabolism. Endogenous L- carnitine is obtained from two essential AAs, lysine, and methionine. However, in humans, 75% of carnitine is obtained from the diet [124]. Additional carnitine is mainly available from animal sources but has limited availability in vegetarian food. The preventive role of L-carnitine and acetyl-L-carnitine (LAC), in neuro-degenerative [125,126] and psychiatric [127,128] disorders is widely discussed. One outcome of the epigenetic perspective is the new possibility for interventions that help the brain and the rest of the body to achieve a successful outcome in the face of adversity (resilience). LAC is a natural molecule that rapidly up-regulates the metabotropic glutamate receptor, mGlu2, via an epigenetic mechanism involving acetylation of lysine 27 on histone H3 (similarly to the histone deacetylase inhibitors, acts in a few days in multiple rodent models of chronic stress [127,128,129,130,131,132]. Recent translational studies also showed decreased LAC levels in clinical endophenotypes of depression. Furthermore, LAC deficiency is associated with both peripheral and central insulin resistance [133,134], which can be ameliorated by supplementation, at least in rodent models of LAC. These findings support the need for the further development of personalized medicine strategies to effectively treat depression. However, despite the known examples of the toxicity D-AAs isomers, the racemization parameters of the stereoselective form of carnitine in the diet and pharmaceutical supplements are not a primary focus of LAC research [129,131]. Experimental evaluation of racemization rate of L-carnitine will significantly contribute to the value of scientific conclusions and suggestions for treatment.

## 3. Universal Biological of Aging

The perceptual and cognitive representations of the external world are associated with the internal events at the different levels of biological organization, including interaction of the CNS with the immune and endocrine systems/At the cellular level, cognitive representations are reflected in the neuronal representations [82,106] that are coordinated with the multitude of biomolecular events. At the molecular level, the fundamental age-related biological clocks are associated with the alterations in the biopolymers, such as DNA, RNAs [134,135,136], lipids [124], proteins [80,81] and interaction between them [137]. All molecular clocks contain chronological and biological information related to the genetic and epigenetic impacts [138]. At the protein level, the age-related changes are documented in protein transcription, translation, and PTMs. At the same time, neuronal representations are linked to the dynamic system of enzymatic PTMs. It is well-documented that the dysregulation of PTMs leads to a range of neurological alterations (including intellectual disability, learning and memory impairments, autistic-like features, and seizures) [81,82,139,140,141,142]. Aberrant PTMs can potentially be used as the biological indicators of the acute as well as the ageing linked psychological and cognitive states. At PTMs domains, the significant age-induced changes are observed in the enzymatic PTMs. Most dramatic age-related changes occur in the level of protein phosphorylation [81]. Notably the enzyme family of serine-threonine protein kinases, known as the major modulators of cellular transformations, target the most racemization-prone AAs residues [34,84]. Therefore, non-enzymatic racemization of serine (Ser) and threonine (Thr) predisposes cell survival functions that are vulnerable to spontaneous time-dependent PTMs. 

Many publications have described the interaction between mental representations, memory functions, and activity-dependent PTMs of proteins as reciprocal. Convincing examples of such mutual impact provide the studies of molecular determinants of age-associated cognitive and mental deterioration in AD (see review [143]). However, the answer to how and to what degree mental determinants of cognitive and psychological states can impact the PTM system requires additional experimental efforts. The convincing example illustrating the potential pathogenic effect of spontaneous racemization is protein phosphorylation. Diverse families of protein kinase are the essential participants of SRS. Functional alterations on kinases directly contribute to age-dependent neuronal loss, finally leading to cognitive decline. The eukaryotic protein kinase (EPK) family is one of the largest protein families in the human genome [144]. The sub-family of ERK Ser/Thr kinases are the critical and well-studied determinants of cell physiology and pathology. The distinct member of the family is the protein complex TORC (in mammalians, TOR (mTOR) exists as a complex of proteins called mTOR complex or mTORC. mTORC operates within two functionally and structurally distinct complexes: mTORC1 and mTORC2. mTORC2 regulates cytoskeletal organization). TORC is evolutionary conserved, and homologs are found in yeast, nematodes, flies, plants, and all mammals. Mammalian TOR (mTOR) signaling has been demonstrated to control almost all fundamental molecular and cellular processes, including protein and lipid synthesis, protein PTMs, DNA modification, autophagy, apoptosis, cell growth, proliferation, and functions [145], Two stereoisomers of phospholipids have separately emerged in archaea and bacteria, an evolutionary divergence known as “the lipid divide” [141].

As a key component of cellular metabolism, mTOR integrates environmental stressors demands), cell physiology, and organism behavior. The molecular mechanism of (mTOR regulates life, including nutrient span, aging, and age-related pathologies [146,147,148]. The amino acids (AAs) sequence of mTORC1 contains functionally essential and racemization prone Ser residues [149]. In addition, downstream EPK signaling occurs through the covalent transfer of the terminal phosphate group from ATP or GTP to Ser, Thr, or tyrosine (Tur) residues of substrate proteins [150]. Reversible phosphorylation of the Ser and Thr modulates conformational switching in regulatory proteins to alter corresponding signaling and transcription [151]. The combination of the regulatory proteins alters corresponding signaling and transcription [151]. The combination of the above two factors provides the high probability of spontaneous age-associated PTMs compromising mTOR function, explaining the known association of mTOR signaling with life span, aging, and age-related pathologies. mTOR is an illustrative example of many proteins containing racemization-prone AA residues, speaking to support the racemization hypothesis of aging.

## 4. Conclusions

Here we provide supporting rationale for the fundamental role of molecular chirality in chirality in the function, aging, and pathology of a human organism. It is not an accidental combination of events that (a) functions of the CNS are based in the molecular chirality [67,152] (b) numerous psychiatric drugs are chiral compounds [153,154,155,156,157], and (c) psychiatric disorders are strongly associated with the bilateral asymmetry of the brain [111,158]. However, the link between these seemingly unrelated events constitutes a challenging barrier for neuroscience and psychology. The most studied dimensions of age and aging are chronological age [159], biological aging (also called physiological aging including molecular and cellular domains) [160], psychological aging [161,162] and social aging [163]. It is widely recognized that “the roots of stress research are based on the belief that stress can accelerate biological aging” [115]. It is also a shared recognition that aging causes decreased resistance to stresses [164] due to the age-associated dysfunction of the SRS [165]. 

Consequently, long-lasting exposure to stress accelerates the aging process [166]. Bidirectional interaction of these two processes results in a self-sustaining mechanism traceable at the cellular and molecular levels. Psychological stressors, linked with the genetic and epigenetic modifications, are known as the influential causal events changing the physiology of many organs of the human body (including brain and gut [167]) and systems (including an immune [168] and hormonal [36]). Aging, accompanied by the decline in health, independence, perceptual, cognitive, and decision-making abilities, is closely associated with the negative impacts of typical psychological stress [6,169,170]. Indeed, both psychological stress and aging show cumulative impact on most physiological systems [171,172,173] involving biological events at the molecular [173], cellular [174] and system [34,41] levels. Epigenetics alterations are triggered by the stressful or beneficial environmental input [175]. Excessive and chronic stresses are associated with accelerated molecular and cellular aging. Time-dependent accumulation of changes is a major molecular determinant of organism aging [174]. It is a common agreement that epigenetic age-associated modifications at the protein level are induced by the spectrum of psychological and biological stressors [175,176,177]. However, the role of prevalent protein chirality in this process is mainly overlooked. The complexity of PTMs is a thermodynamically driven mechanism to convert the epigenetic signals (stressors) into biological responses implemented by the SRS.

The chain of interlinked molecular, cellular, systemic and organism level of biological events allows consideration of non-enzymatic racemization, time-dependent and irreversible (under physiological conditions, i.e., relatively irreversible) PTMs as the fundamental determinant of protein aging and biological age of the organism. Experimental evidence shows that αA-crystallin (small heat shock protein), containing about 30% of intrinsically disordered residues [178], is undergoing age-related racemization at Ser residues [179]. This fact suggests that both globular and intrinsically disordered proteins (IDPs), despite the significant difference in their configurational entropy and energy landscape [180], are prone to the impact of spontaneous racemization. An organism’s aging and its counterpart spontaneous molecular events are inevitable processes. However, this body of evidence suggests that epigenetic aging could be if not preventable than treatable for therapeutic intervention [159,181,182,183,184,185]. Ribosome-mediated translation machinery (TM) of the cell incorporates only L-AAs in the chain of synthesized proteins [186]. The essential advantages attributed to protein homochirality are clearly articulated [187,188]. A homochiral chain of AAs (characterizes by a high level of entropy) is more prone to spontaneous condensations and secondary structure formation (such as the formation of α-helices and β-sheets) than the heterochiral or racemic variants. The chain of spontaneous condensations lowers the number of accessible states to the folded protein, reducing entropy’s translational, configurational, rotational, and vibrational components. (In the cytosol or lipid environment the high entropy of the newly synthesized homochiral chain of L-AAs (state I) is spontaneously collapsing to the low value (state II) (accompanied by increased solvent entropy) and spontaneously returning to the high level of in the denaturalizing (non-functional) conformations (state III). One of such denaturalizing conformations is the racemization of AAs. In both conditions, the nascent homochiral AAs chain and corresponding racemic variant are characterized by the high entropy relative to the native state. The critical difference is that the newly synthesized AAs chain is in the non-equilibrium state, while the racemic variant is in equilibrium.) 

Physiologically relevant compact structures (NSs) are occurring by the spontaneous lowering entropy of the initial AAs chain accompanied by increased local inter-residue interaction to ensure protein stability (lowering the energy barrier and energy minimum). The native states, being in the non-equilibrium condition, are prone to the second round of PTMs SP–racemization accompanied by increased entropy.

In the recent decade, significant advances achieved in understanding the fundamental role of protein chirality. First, D-AAs are found in various living higher organisms in the free form and the bound state in peptides and proteins [189]. Second, the contribution of protein to organism aging is associated with AAs’ susceptibilities to non-enzymatic PTMs [190]. Third, the age-associated chain-specific “collapse of homochirality” of AAs in proteins is observed in the human body’s various tissues and organs (including the brain) (recent advances in chirality transformation in isotropic systems reveal the possibility of two modes of relevance for mirror symmetry breaking with the low- and high-energy of the racemic ground state [191] (terminology introduced by Fujii) [192].

To better understand advanced biological aging, it is necessary to examine the interrelation of molecular determinants of the biological clock with the somatic and mental health conditions [193,194,195]. Cross-talk of biological, cognitive, and psychological clocks provides an integrative contribution to healthy and advanced aging [162,194,195]. However, the fundamental (i.e., essential for all levels of biological organization) role of spontaneous PTMs in organism aging and dysfunctions is currently not targeted by systematic proteome-wide exploration.

## Data Availability

Not applicable.

## Abbreviation

AAs	Amino acids
ATP	Adenosine triphosphate
D-AAs	D-amino acids
AD	Alzheimer disease
A-b	Amyloid-beta
LAC	Acetyl-L-carnitine
DNA	Deoxyribonucleic acid
EPK	Eukaryotic protein kinase
LLPs	Long-lived proteins
mTOR	Mammalian TOR
MA	Mutation accumulation theory
AP	hypotheses. Antagonistic pleiotropy
NS	hypotheses. Native state
NS	Natural selection
NE	Non-equilibrium
NTs	Nucleotides
PTM	Post-translational modification
RHA	Racemization hypothesis of aging
RNAs	Ribonucleic acids
PTMs SP	Spontaneous PTMs
Ser	Serine
SRS	Stress response systems
Tyr	Tyrosine
